# Video evidence and other information relevant to the conservation of the Ivory-billed Woodpecker (*Campephilus principalis*)

**DOI:** 10.1016/j.heliyon.2017.e00230

**Published:** 2017-01-24

**Authors:** Michael D. Collins

**Affiliations:** Naval Research Laboratory, Washington, D.C. 20375, USA

**Keywords:** Ecology, Zoology

## Abstract

Despite the publication of three independent papers that reported sightings of the Ivory-billed Woodpecker (*Campephilus principalis*), its preservation became a controversial topic because nobody has been able to obtain a distinct photo, which is considered the standard form of evidence for documenting birds. An analysis based on a combination of factors related to habitat and behavior suggests that the expected waiting time for obtaining a distinct photo of an Ivory-billed Woodpecker is several orders of magnitude greater than it would be for a more typical baseline species of comparable rarity. Given these difficulties and the time pressure involved to ensure the conservation of this species, this paper discusses the need to use a different approach for an exceptional case. Presented here are three videos that show birds with flights, behaviors, field marks, and other characteristics that are consistent with the Ivory-billed Woodpecker but no other species inhabiting the region.

## 1 Introduction

The Ivory-billed Woodpecker (*Campephilus principalis*) is the only North American bird that has been thought to be extinct only to be rediscovered multiple times [1, 2], but it has not been photographed for the past several decades. After discovering a remnant population of Ivory-billed Woodpeckers in Cuba in 1948 [3], John Dennis continued searching and reported three encounters with these birds during the next twenty years [4]. On the basis of his experience in the field, Dennis made the following comments about searching for the Ivory-billed Woodpecker in the swamp habitats in the mainland part of its range (which have little in common with the highland habitats of the Cuban subspecies) [5]:

“It takes a couple of years to search out and find the Ivorybill in only a single swamp.”

“It is next to impossible to obtain photographs of an Ivorybill in a southern swamp unless a nesting site is discovered.”

In recent years, numerous sightings were reported during independent search efforts in Arkansas [2], Florida [6], and Louisiana [7], but indisputable photographic evidence is unfortunately lacking. This paper presents an analysis of the expected waiting time for obtaining such evidence and video footage suggesting that this reclusive species may still persist in North America.

## 2 Materials and methods

### 2.1 Model for the expected waiting time for obtaining a photo

The elusiveness of the Ivory-billed Woodpecker may be approximately quantified in terms of a set of factors related to habitat and behavior relative to a more typical baseline species of comparable rarity that resides in a baseline habitat. I consider a simple model in which a single Ivory-billed Woodpecker is present in the habitat. In this model, the expected waiting time for obtaining a photo,

(1) $$E=\frac{A}{B}\;\;\;\sigma \;\;\;,$$

is assumed to be directly proportional to the area A of the habitat and inversely proportional to the net area B that is searched per unit time along all search paths, where the proportionality factor σ depends on habitat and behavior. A single individual of the baseline species is assumed to be present in the baseline habitat, and the expected waiting time for obtaining a photo is

(2) $$E_{0}=\frac{A_{0}}{B_{0}}\;\;\;\sigma_{0}\;\;\;\;\;,$$

(3) $$\frac{E}{E_{0}}=\frac{A}{A_{0}}\;\;\;\frac{B_{0}}{B}\;\;\;\frac{\sigma }{\sigma_{0}}\;\;\;,$$

where $A_{0}$, $B_{0}$ and $\sigma_{0}$ are the corresponding quantities for the baseline case.

### 2.2 The fieldwork and analysis of video evidence

Between November 2005 and June 2013, I spent several months per year searching for Ivory-billed Woodpeckers. Most of this work was conducted in the Pearl River swamp in Louisiana, which has a history of reported sightings of these birds. I spent additional time in the Choctawhatchee River swamp in Florida [6]. The search was conducted on an opportunistic basis and involved approximately 500 visits ranging from a few to several hours, resulting in approximately 1500 hours of observation time. The frequency of visits ranged from sporadic during the warmer months to several times per week during the winter months, which are generally believed to be more favorable for searching for these birds. Most of the visits were in the early morning or late afternoon, when birds tend to be more active. Most of the visits to the Pearl River swamp were between Interstate 10 and Old Highway 11. Movie S1 shows part of this area and locations where video evidence was obtained.

**Fig. 1:**
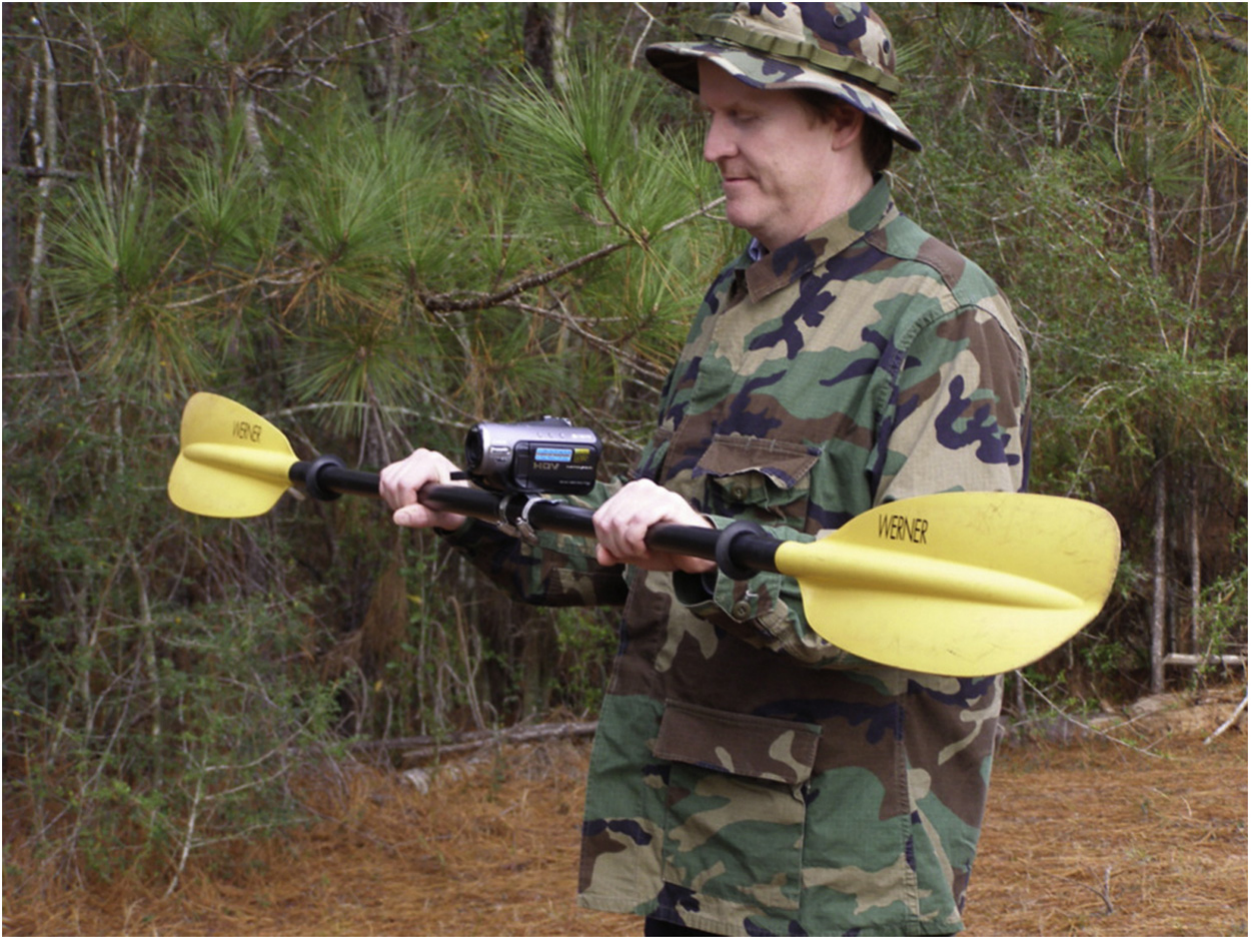
High-definition video camera mounted on kayak paddles used in this study. The focus is set to a moderate range (such as 50 m), and the camera is kept recording at all times. With this set up, the paddles may be used to get the camera on a bird almost instantly as illustrated in the example in Movie S2.

Much of the search was conducted using a kayak, an approach that had been found to be effective in Arkansas [2]. In heeding reports of the wariness of Ivory-billed Woodpeckers, I avoided pursuing the birds after sightings. After having sightings of birds that flushed from near the bank of the bayou and rapidly flew into cover, I was motivated to mount a high-definition video camera on the kayak paddles as shown in Fig. 1. The camera is kept recording at all times, and the kayak paddles are used to quickly get the camera on birds as illustrated in Movie S2. A second approach employed was to use exceptionally tall trees as observation platforms, aiming to observe Ivory-billed Woodpeckers flying over the treetops in the distance. A similar approach was used in Arkansas [2], but it involved the use of a crane rather than trees.

The 2006 and 2008 videos were obtained using a Sony DCR-HC36 standard video camera. The 2007 video was obtained using a Sony HDR-HC3 high-definition video camera. The videos were analyzed using inputs from ornithologists and avian artists [7], published results on the flight mechanics of birds [8, 9, 10], historical accounts of the flights and behaviors of the Ivory-billed Woodpecker, historical photos and film of Ivory-billed Woodpeckers, a film of the closely-related Imperial Woodpecker (*Campephilus imperialis*) [11], a Pileated Woodpecker (*Dryocopus pileatus*) specimen that was mounted on part of a perch tree that was collected, and video footage of Pileated Woodpeckers.

## 3 Results and discussion

### 3.1 Factors that affect the expected waiting time for obtaining a photo

I discuss here factors related to habitat and behavior that affect the expected waiting time for obtaining a distinct photo of an Ivory-billed Woodpecker. These birds reside in the interior of vast flooded forests. The drone video footage in Movie S1 gives an impression of the vastness of the Pearl River swamp, even though it shows only a small fraction of it. There is cover in all directions in the interior of a forest, which limits visibility to small areas, provides many hiding spots for a wary bird, and makes photography more difficult. Both the size of the habitat and the limited visibility significantly reduce the likelihood of discovery.

Through the combined efforts of a large community of bird watchers, convincing photographic evidence has been obtained for many rare birds, but the Ivory-billed Woodpecker resides in habitats that most bird watchers never visit. The interior of a swamp forest’s low species diversity does not attract bird watchers [12], and numerous deterrents may keep them away. It is physically demanding to penetrate deeply into such habitats; alligators, wild boars, and venomous snakes are abundant; and there is the possibility of heat stroke during the hot and humid summers and hypothermia during the cold and damp winters. Strong currents, rapidly rising water, and heavy hunting activity make swamp forests dangerous. On the basis of discrepancies in the *Florida Breeding Bird Atlas* and the lack of any sign of visitors during his first year of fieldwork, Hill concluded that bird watchers rarely visited the Choctawhatchee River swamp before his findings were made public [12]. During eight years of fieldwork, I only occasionally saw bird watchers along a paved road that provides access to a small percentage of the Pearl River swamp but never encountered them in the remote areas miles from the road where the videos presented here were obtained. Even those who are not deterred from entering a swamp forest find it difficult to move along a search path and adequately cover all parts of the habitat. There are flooded areas where one may sink deep into the mud with every step, networks of bayous that impede access to some areas by foot, and areas that are clogged with thick vegetation and the aftermath of hurricanes through which it is difficult to approach a wary bird without being detected.

A lack of conspicuous behaviors can have a profound effect on the expected waiting time for detecting a bird. For example, let us consider an acre of flatwoods pine habitat in Florida, where a Blue Jay (*Cyanocitta cristata*) and a Bachman’s Sparrow (*Aimphila aestivalis*) are present during the non-breeding season, with an experienced bird watcher passively observing from the boundary of the area. It is likely that the jay would be detected almost immediately, but the sparrow could go undetected for several days. The Ivory-billed Woodpecker lacks conspicuous behaviors according to Allen and Kellogg [1], who made the following comments on the birds they observed in the Singer Tract:

“We had hunted for three days for this particular pair of birds without ever hearing them, even though we were frequently within three hundred yards of the nest, which we finally found because we happened to be within hearing distance when the birds changed places on the nest.”

“They are not noisy except when disturbed.”

“Their voice does not carry nearly as far as that of the Pileated Woodpecker.”

“In the big trees which they normally frequent they are easily overlooked.”

“We camped for five days within three hundred feet of one nest and, except when the birds were about to change places on the nest or were disturbed, seldom heard them.”

The birds that were studied near the last known nest sites in the Singer Tract became acclimated to the presence of humans [1, 13], but the behavior of those birds is of little relevance to the expected waiting time for obtaining a photo without a previously known nest site. The Ivory-billed Woodpecker is an exceptionally wary species according to John James Audubon and Arthur T. Wayne [13] (p. 63), and their accounts from the 19th Century are consistent with numerous reports during the past several decades. A bird that lacks conspicuous behaviors, such as soaring above, using a prominent perch, or making frequent vocalizations that can be heard from a distance, may not be detected until there is a close encounter, and there may be many near misses along the search path (e.g., see the first comment above by Allen and Kellogg). If the bird is also wary, it may move away from the search path before it can be detected, and it will rapidly seek cover when flushed.

Since Ivory-billed Woodpeckers fly long distances to forage [1, 13], most sightings are likely to be far from a nest or roost cavity. Ornithologists were already aware by the 1930s that this non-territorial behavior accounts for sporadic sightings in which the birds could not be relocated [14]. When a non-territorial and exceptionally wary bird is flushed at a location far from a nest or roost, there may be only one chance that lasts for only a few seconds to obtain a photo. If the opportunity is missed, it might take years of searching before another opportunity arises. The camera must be kept ready for encounters that do not allow time to maneuver into a position that is favorable for obtaining a photo. In addition to the regular flights to foraging areas, Ivory-billed Woodpeckers are believed to make longer-term, nomadic moves in response to the waxing and waning of favorable food sources, such as after a hurricane or a drought that kills a large number of trees. In a report of a study in the Singer Tract, for example, Richard Pough [15] noted that the habitat became favorable for Ivory-billed Woodpeckers during a major drought in the 1920s and that the birds actually began to disappear from the area as the food sources waned before logging commenced. There have been no recent reports from the sites where the searches in Arkansas, Florida, and Louisiana took place. Ivory-billed Woodpeckers could be absent from sites that seem favorable but present at sites that have been overlooked.

I define a baseline case in order to apply the model. The baseline habitat would be small enough to be thoroughly searched in one day; visibility would be good out to long distances in all directions; it would be easy to follow a search path; and there would be regular visits by a substantial number of bird watchers. The baseline species would have conspicuous behaviors that make it easy to find and photograph; be sufficiently non-wary to remain in the area after a sighting and to allow a close enough approach to obtain a distinct photo; have a territory that is small enough so that it would be easy to relocate a bird after an initial sighting; and reside in the same areas for many generations.

Since Ivory-billed Woodpeckers typically reside in habitats that cover more than $100{\;\;\;\text{km}}^{2}$, it would be challenging to thoroughly search the baseline habitat in one day even if we take $A/A_{0}=100$. Substituting into the model for this case, I obtain

(4) $$\frac{E}{E_{0}}=10^{2}\;\;\frac{B_{0}}{B}\;\;\frac{\sigma }{\sigma_{0}}\;\;\;.$$

Visibility typically starts becoming limited beyond ranges of a few tens of meters in a forest, even when the leaves are down in the winter. A large bird with prominent field marks, such as an Ivory-billed Woodpecker, could be detected and identified with binoculars out to hundreds of meters in the baseline habitat. Since the ratio of distances is about 10, the ratio of areas is about 100, and we would obtain $B_{0}/B≅100$ if bird watchers covered the same net distance per unit time along all search paths in both habitats. If the coverage by bird watchers is actually much greater in the baseline habitat, then $E/E_{0}$ is much greater than $10^{4}\sigma /\sigma_{0}$. The proportionality factors depend on the degree to which the species are wary, conspicuous, territorial, and nomadic and the difficulty of moving along search paths in the habitats. The above discussion of these factors suggests that $\sigma /\sigma_{0}$ is large and that the expected waiting time for obtaining a distinct photo of an Ivory-billed Woodpecker is several orders of magnitude greater than it would be for a more typical baseline species of comparable rarity.

### 3.2 Video evidence from Louisiana

During a five-day period in February 2006, I had five sightings and twice heard the characteristic ‘kent’ calls of the Ivory-billed Woodpecker in a concentrated area along English Bayou in the Pearl River swamp [7]. During a sighting on February 16, the bird flushed from close range on the bank with wingbeats that seemed unusually rapid for such a large bird and provided an excellent view of the definitive field marks on the dorsal surfaces of the wings. During a sighting on February 17, the bird glided low across the bayou and provided a clear view across the dorsal surfaces of the wings. The characteristics of the flights that were observed are consistent with historical accounts by Tanner and Audubon and similar to the characteristics of flights that appear in the videos. On February 18, I heard a long series of kent calls while drifting down the bayou in a kayak and quietly approached to within perhaps 5 m of the bird, which was behind a fallen tree on the bank. Kent calls then started coming from a second bird on the opposite side of the bayou. After the second bird apparently noticed me near the first bird, a few harsh calls came from the direction of the second bird, and the first bird went silent. The harsh calls were consistent with a scolding call that Allen and Kellogg [1] observed when a female Ivory-billed Woodpecker became alarmed near a nest. The harsh calls were followed by a series of high-pitched calls that came from the same direction. On February 19, I returned to the area with a video camera but did not detect the birds.

On February 20, I had a sighting in the area where the calls came from the second bird on February 18. After the bird flew into the woods, the same high-pitched calls started coming from that direction. The video camera was turned on, and several of the calls were captured in the audio track of the 2006 video. They seem to be consistent with an account by Tanner [13] (p. 62) of high-pitched calls that are given when an Ivory-billed Woodpecker is alarmed. They have the same type of sonogram structure as kent calls, with both calls consisting of simultaneously excited harmonics [7]. After the high-pitched calls stopped, I backed the kayak into an observation position on the opposite bank. About 10 min into the video, motion was detected in a tree deep in the woods on the opposite side of the bayou, and footage was obtained of a large woodpecker that has several characteristics consistent with an Ivory-billed Woodpecker but not a Pileated Woodpecker. The field marks, body proportions, and other characteristics of birds are studied in great detail by avian artists, such as David Sibley, who provided comments on a video that was obtained in Arkansas [16]. After studying the video that was obtained in Louisiana in 2006, Julie Zickefoose, an artist whose paintings of Ivory-billed Woodpeckers have appeared on the covers of an ornithology journal (the January 2006 issue of *The Auk*) and a book on this species [17], remarked on the “rared-back pose, long but fluffy and squared-off crest, and extremely long, erect head and neck” [7]. The bird in the video required a deep and rapid flap to cover a distance of less than 1 m during a short flight between limbs. The Ivory-billed Woodpecker usually flaps its wings during short flights between limbs according to Tanner [13] (p. 58). This would make sense for a woodpecker that is one of the most massive in the world and has narrow wings that are adapted for long flights at high speed. The Pileated Woodpecker has a much lower mass and broader wings that are adapted for the short flights of a territorial species, and it frequently makes such flights nearly effortlessly. Movie S3 contrasts the short flight by the large woodpecker in the video with short flights by Pileated Woodpeckers.

**Fig. 2:**
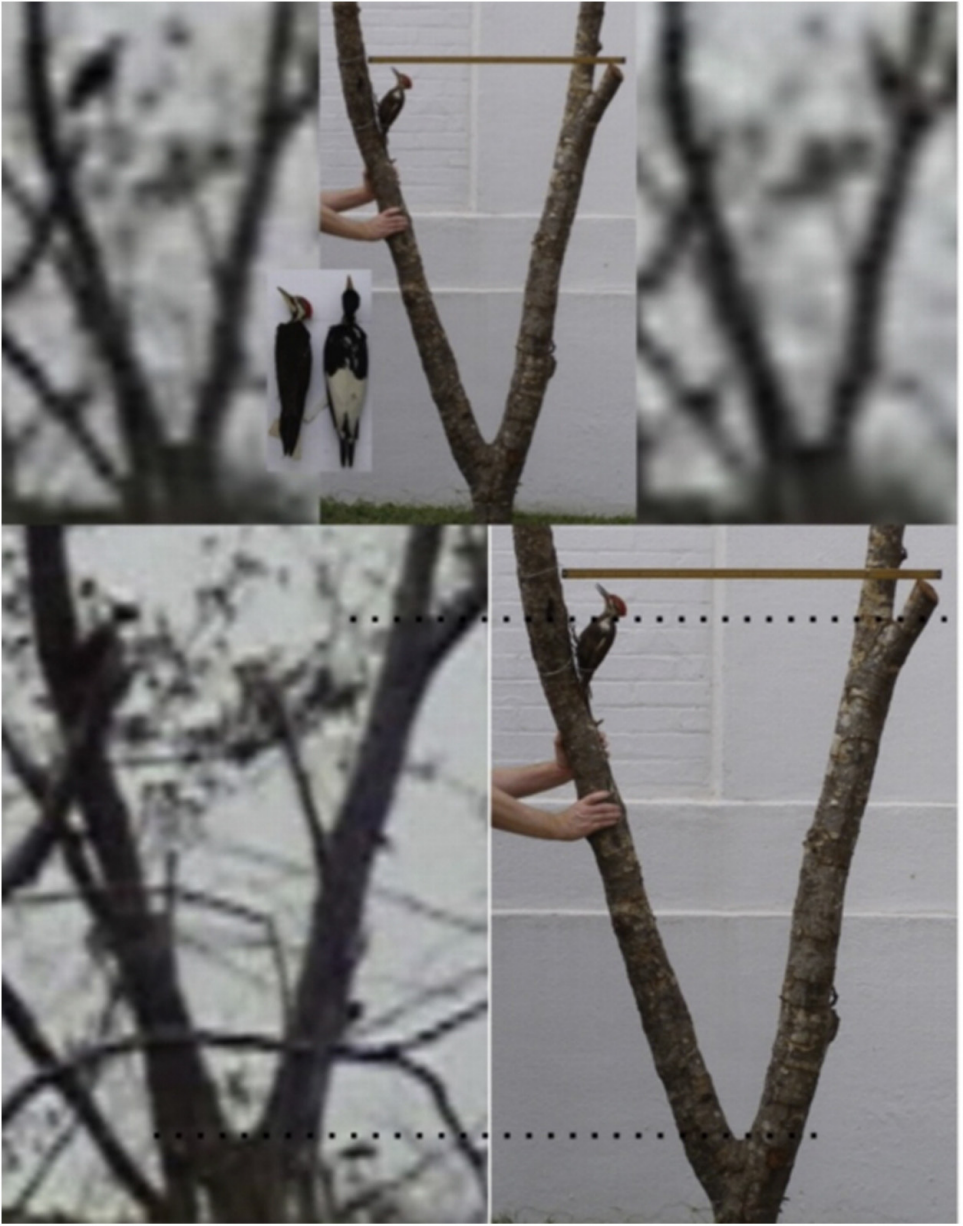
A Pileated Woodpecker specimen is mounted on part of the perch tree. Frames from the 2006 video [7], part of which appears in Movie S3, were scaled using forks in the tree (dashed lines). A meter stick is placed at the point where the flight between limbs occurred. The inset shows Pileated Woodpecker and Ivory-billed Woodpecker specimens that were photographed side by side. The bird in the video is partially hidden by vegetation in the image on the lower left, but it is fully in view in the images at the top when it took the flight between limbs.

Part of the perch tree, which was collected after it blew down in the summer of 2008, was used along with a Pileated Woodpecker specimen for the size comparison in Fig. 2. As can be seen from the specimens that appear in the inset photo, the Ivory-billed Woodpecker is larger than the Pileated Woodpecker. The bird in the video is partially hidden in one of the images, but it is clear from the other images that it is a large woodpecker, and, in fact, it appears to be larger than the mounted Pileated Woodpecker specimen and consistent in size with an Ivory-billed Woodpecker. A laser rangefinder was used to determine that the distance from the observation position to the perch tree was 128 m. As illustrated by the examples in Movie S4, I frequently observed Pileated Woodpeckers that showed no concern for my presence at ranges of a few tens of meters. In 1892, Arthur T. Wayne observed Ivory-billed Woodpeckers that were too wild to be “approached nearer than 300 or 400 yards” [13] (p. 63). The large woodpecker in the video was well beyond the range at which a Pileated Woodpecker would become alarmed but well within the range at which an Ivory-billed Woodpecker would become alarmed according to Wayne’s account. It showed signs of being alarmed, including raising its crest and hiding behind a large branch. The encounter began at about 7:25 a.m., when a non-alarmed Pileated Woodpecker would be actively going about its business foraging, calling, and drumming, but the large woodpecker in the video never engaged in any of those behaviors. No calling or drumming by a Pileated Woodpecker was captured in a continuous stream of 37 min of video.

On March 29, 2008, I was keeping watch out over the treetops from 23 m above the bayou in a cypress tree, less than 1 km from the site where the video was obtained in 2006, when an Ivory-billed Woodpecker flew up the bayou and passed nearly directly below. I observed the definitive dorsal stripes and field marks on the dorsal surfaces of the wings from a favorable vantage point at close range. The video that was obtained during the encounter appears in Movie S5 and is discussed in Movie S6. It is not generally possible to determine the position of an object that appears in a video. Since the bird and its reflection from the surface of the bayou are visible in the video, however, it was possible to determine positions of the bird along its flight path, which were marked with stakes as discussed in Movie S6, and estimate the flight speed [7]. Since the bird was initially flying nearly directly toward the camera, the video simultaneously shows the two components of wingtip motion that are used in an approach for analyzing woodpecker flight mechanics that was developed by Tobalske [8], who digitized the wingtip motion from the video and concluded that the bird in the video is a large woodpecker [7].

As discussed in Movie S6, the bird in the video folds its wings closed during the middle of each upstroke. The two large woodpeckers are the only large birds of the region with that distinctive wing motion during cruising flight (all of the others keep their wings open throughout the flap cycle). This type of wing motion is shown for a Pileated Woodpecker in Movie S7. Based on observations in the Singer Tract, Eckleberry described a “flight in which there seemed to be very little movement of the inner wing” [18]. The wing motion that Eckleberry reported is not at all like the wing motion of the birds in Movies S5 and S7, but his observations were of a bird that was approaching a roost tree and may have been in a flap-glide flight just before landing. The bird in the video was in cruising flight, a well-studied type of avian flight that is known to be amenable to statistical analysis [9]. The Ivory-billed Woodpecker has a high flap rate according to Tanner [13] (p. 58), and the narrow wings, high mass, and high flight speed of this species correlate with a high flap rate according to flap rate models [9, 10]. Tanner’s account would only make sense if it were a tacit comparison of the flap rates of the two large woodpeckers. It seems unlikely that such a comparison could have been made reliably based entirely upon observations in the field (no flights appear in the historical film) unless there is a substantial difference between the flap rates of the two large woodpeckers. Published values for the cruising flight parameters of the Pileated Woodpecker include a mean flap rate of 5.2 Hz, standard deviation of 0.4 Hz, and flight speed of 7.5 to 11.6 m/s [8] (the values for the flap rate statistics are consistent with the flight in Movie S7 and other data that were obtained during the fieldwork in the Pearl River swamp). The bird in the video has a flap rate of about 10 Hz and a flight speed of about 15.2 m/s [7]. As discussed in Movie S6, the bird in the video has narrow wings, swept back wings, and white patches on the dorsal surfaces of the wings, all of which are consistent with an Ivory-billed Woodpecker but not a Pileated Woodpecker.

### 3.3 Video evidence from Florida

On January 19, 2007, I had a sighting during a visit to Hill’s study area in the Choctawhatchee River swamp. I was participating in a follow up to a report the previous day by a member of Hill’s search team in which the birds left the area when the observer attempted to approach them. I came upon the birds while kayaking along a small bayou with the high-definition video camera mounted on the paddles. The definitive field marks on the dorsal surface of the right wing of one of the birds were observed through binoculars, and a series of acrobatic swooping flights that were observed immediately brought to mind Audubon’s account of a flight that is “graceful in the extreme” [14]. I did not attempt to approach the birds and was able to record several events with the paddle-cam, including some of the swooping flights. In the discussion of events, times are noted in minutes and seconds from the beginning of the video.

An event that began at 17:44 is shown in Movie S8. The bird climbed upward, moved to the right, moved back to the left, perched upright, delivered a blow that produced an audible double knock (with a slight delay due to the distance to the bird), and then took off into an upward swooping flight (the audio contains dialogue with a member of Hill’s search team who arrived on the scene during the encounter). The sound of the double knock is suggestive of a blow by a large woodpecker. Ivory-billed Woodpeckers are known to give single and double knocks as signals [13] (p. 62), but this behavior is not consistent with any of the other woodpeckers of the region. There was no drumming either before or after the double knock. After drumming, a woodpecker typically remains perched and listens for a response, but the bird took off immediately after delivering the blow. The takeoff that immediately transitions into a high-speed upward swooping flight does not seem to be consistent with any of the other woodpeckers of the region, but the Ivory-billed Woodpecker is known from historical accounts to have remarkable flights. When delivering the blow, the bird appears to have white on the belly, but it can be seen that this is vegetation in the foreground by noting the bird emerging from behind it before moving to the right. There does not exist an undisputed recording of a double knock of an Ivory-billed Woodpecker. No information is available on the time interval between knocks for this species, the variability of this quantity, and how it compares to the same quantity for other *Campephilus* woodpeckers.

The Ivory-billed Woodpecker has a much greater body mass than the Pileated Woodpecker, and its close relative, the Imperial Woodpecker, is even larger. When these massive woodpeckers move around in a tree, it would make sense that they would need to flail their wings for balance more frequently than the Pileated Woodpecker and other smaller woodpeckers of the region. This is a common behavior of the Ivory-billed Woodpecker according to Tanner [13] (p. 58), who described it using the phrase, “flirting the wings.” As discussed in Movie S9, an Imperial Woodpecker flailed its wings in the only 85 s of film that exist for that species [11], and the bird in the video flailed its wings several times just before delivering the blow. The series of occurrences of this behavior does not seem to be consistent with other woodpeckers. As discussed in Movie S10, Ivory-billed Woodpeckers in the historical film nearly constantly rotate their bodies from side to side while perched on their tails. Other woodpeckers occasionally make these motions at certain locations (such as a nest or drumming site) but do not seem to engage in this behavior while moving around and foraging. As discussed in Movie S10, the bird in the video made these motions at two points during the event.

Movie S11 shows a takeoff into a level, non-escape flight at 5:36 of a bird that appears to be large on the basis of its size relative to the substantial broken-off tree and appears to be a woodpecker on the basis of the way it hopped behind the tree at 5:14 as shown in Movie S12. Prior to hopping, the bird was sitting across a limb, a behavior that is consistent with an account by Tanner [13] (p. 57). Movie S11 also shows takeoffs into level, non-escape flights by an Imperial Woodpecker [11] and Pileated Woodpeckers. The deep and rapid flaps of the bird in the video do not seem to be consistent with the Pileated Woodpecker, but they are similar to the deep and rapid flaps of the Imperial Woodpecker and consistent with the Ivory-billed Woodpecker in terms of accounts by Tanner [13] (p. 58) of “particularly hard” flaps at takeoff and Christy [19] of “deep and rapid strokes” at takeoff.

Movie S13 shows an unusual upward swooping landing at 5:10. As discussed in Movie S14, the bird appears to have a black body (including the belly) and a right underwing that is mostly white. The two large woodpeckers are the only candidate species with those field marks. The bird ascended nearly vertically without flapping for about 1 s, which would correspond to about 5 m for a ballistic flight and an even greater vertical distance for a swooping flight that ends with braking. This remarkable flight does not seem to be consistent with the Pileated Woodpecker, but it is consistent with Audubon’s account of the Ivory-billed Woodpecker having a flight that is “graceful in the extreme” [14]. Movie S15 shows a vertically ascending landing at 15:21. It appears that the view is from the side at the beginning of the event, but the mostly white undersides of the wings are visible when the bird is near the top edge of the field of view. It appears that the bird rotated about its main axis during the ascent. Most woodpeckers swoop upward a short distance before landing on a surface that faces the direction of approach. A long vertical ascent opens up the possibility of rotating about the axis and landing on a surface that faces in any direction. Movie S16 shows a vertically ascending landing at 5:44.

Movie S17 shows a downward swooping takeoff at 24:37 that is viewed from the side. After briefly going below the field of view, the bird reappeared in a ventral view of an unusual landing that is discussed in Movie S18. As in the landing at 5:10, the bird ascended nearly vertically for a long distance and apparently rotated about the axis. In both cases, the view was initially from the side but shifted to ventral before the landing. This could have been a coincidence, but the initial orientation would provide a wary bird with a view of me during the flights and rotating to the other orientation would allow landings on surfaces that were not visible to me. Just before the landing, there is a frame that reveals a dark-colored belly, light-colored underwings, a tail that projects behind the wings about the same distance as the width of the wings, and a body that has a width that is a substantial fraction of the width of the wings. This combination of characteristics is not consistent with any woodpecker of the region other than the Ivory-billed Woodpecker. When an Ivory-billed Woodpecker is perched with the wings folded closed, the white trailing edges on the dorsal surfaces of the wings form a white triangular patch. As discussed in Movie S19, there are flashes of white consistent with this field mark when the bird was climbing after the landing.

**Fig. 3:**
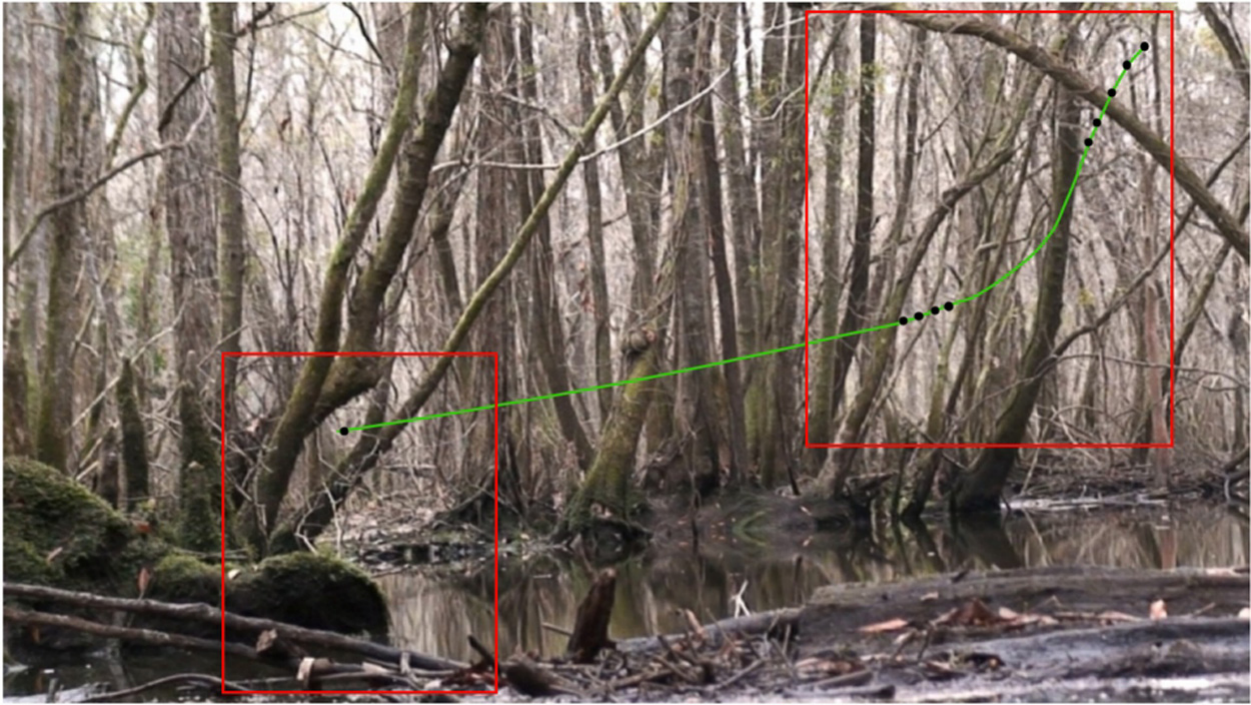
Flight path (green curve) of the swooping takeoff at 2:02 in the 2007 video. The bird was far behind the trees in the foreground. It came in and out of view through gaps in the vegetation (black dots). Movies S20 and S21 were cropped from the video in the areas of the red boxes.

The flight path shown in Fig. 3 corresponds to a swooping takeoff at 2:02 that is similar to the swooping takeoff at 24:37, but in this case the bird leveled off into a long horizontal glide that is consistent with the following comment by Audubon on the Ivory-billed Woodpecker [14]: “The transit from one tree to another, even should the distance be as much as a hundred yards, is performed by a single sweep, and the bird appears as if merely swinging from the top of the one tree to that of the other, forming an elegantly curved line.” The bird was hidden behind vegetation along much of the flight path, but it appears at various points in Movie S20. There is a flash of white from the underwings in the reflection from the water in Movie S21.

## 4 Conclusions

It seems unlikely that unquestionable photographic evidence will be obtained in time to ensure the conservation of the Ivory-billed Woodpecker, should it still exist as the current paper suggests. The video footage presented here shows flights, behaviors, field marks, and other characteristics that are consistent with this species but no other species. While there may still be a chance to save the Ivory-billed Woodpecker from extinction, there is a need to recognize that this species is an exceptional case. Conservation programs are essential to the survival of critically endangered species, as exemplified by the preservation of Kirtland’s Warbler (*Setophaga kirtlandii*), the California Condor (*Gymnogyps californianus*), and the Whooping Crane (*Grus americana*), which might be extinct by now if threats to those species had not been addressed under programs that were established decades ago. The standard approach for documentation is appropriate for those conspicuous birds; however, more than a decade has passed since the most recent rediscovery of the Ivory-billed Woodpecker [2], and due to the controversy regarding the required evidence, no effective conservation program exists for this iconic species. There may still be time to develop a more pragmatic alternative approach for documentation that would allow the allocation of funds for the long-term and widespread protection of the habitat of this nomadic species.

## Declarations

### Author contribution statement

Michael Collins: Conceived and designed the experiments; Performed the experiments; Analyzed and interpreted the data; Contributed reagents, materials, analysis tools or data; Wrote the paper.

### Funding statement

This research did not receive any specific grant from funding agencies in the public, commercial, or not-for-profit sectors.

### Competing interest statement

The author declares no conflict of interest.

### Additional information

No additional information is available for this paper.

## Appendix A Supplementary data

Movie S1 This movie shows the locations where I had sightings and obtained video evidence in 2006 and 2008 in the Pearl River swamp. The video footage that was obtained from a drone in July 2016 illustrates the vastness of the habitat and the density of the vegetation.

Movie S2 Paddle-cam video footage that was obtained in the Pearl River swamp in January 2010. The camera is kept recording at all times, and it is possible to aim the camera with the kayak paddles and get it on a bird almost immediately.

Movie S3 Comparison of the short flight by the large woodpecker in the 2006 video with video footage of typical short flights by Pileated Woodpeckers that were obtained in the Pearl River swamp in March 2006. This footage plays at half speed.

Movie S4 Video footage of Pileated Woodpeckers that was obtained in the Pearl River swamp in January 2007 from within a few tens of meters of the birds, which showed no signs of wariness.

Movie S5 The 2008 video after deinterlacing. This footage plays at full speed and is sampled at 60 frames per second.

Movie S6 Discussion of key aspects of the 2008 video (Movie S5), including the distinctive wing motion of a large woodpecker, comparison of the swept back wings with a historical photo, the narrow shape of the wings, the prominent white patches on the dorsal surfaces of the wings, and how the flight speed was estimated.

Movie S7 A Pileated Woodpecker that was filmed in flight from about 24 m above the ground in one of the observation trees in the Pearl River swamp in February 2015. Note that the wings are folded closed during the middle of each upstroke. The two large woodpeckers are the only large birds of the region with this distinctive type of wing motion. This footage plays at half speed and is sampled at 60 frames per second.

Movie S8 An event beginning at 17:44 in the 2007 video that involved a double knock (that is visible and audible but good speakers and high volume may be required to hear it) and three other behaviors that are consistent with the Ivory-billed Woodpecker but are not consistent with any other woodpecker of the region. This footage plays at full speed.

Movie S9 Discussion of wing flailing behavior that appears in the 2007 video and in a film of an Imperial Woodpecker [11].

Movie S10 A discussion of side-to-side motions that occur at two points in the 2007 video, including a comparison with a common behavior of the Ivory-billed Woodpecker that appears in the historical film. This footage plays at full speed.

Movie S11 A level takeoff into a non-escape flight by what appears to be a large woodpecker at 5:36 in the 2007 video. The deep and rapid flaps are similar to the deep and rapid flaps of an Imperial Woodpecker taking off [11] but quite different from the flaps during typical takeoffs by Pileated Woodpeckers into non-escape flights. This footage plays at full speed.

Movie S12 About 22 s before the takeoff at 5:36 in the 2007 video, the bird was briefly perched across a limb and then it hopped behind the tree. This event is compared with an Ivory-billed Woodpecker hopping into a cavity in the historical film. This footage plays at full speed.

Movie S13 An upward swooping landing at 5:10 in the 2007 video. The long vertical ascent does not seem to be consistent with other woodpeckers of the region. This footage plays at full speed. The effects of camera motion have been reduced using stabilization software.

Movie S14 Discussion of an upward swooping landing at 5:10 in the 2007 video. The bird ascended nearly vertically for about 1 s. It appears to have a black body (including the belly) and underwings that are mostly white.

Movie S15 A vertically ascending landing at 15:21 in the 2007 video. The view appears to be from the side when the bird first comes into view. The view appears to be of the undersides of the wings (which appear mostly white) just before the bird goes out of view at the top edge of the picture (to see this, it may be necessary to go through the video frame by frame). This footage plays at full speed.

Movie S16 A vertically ascending landing at 5:44 in the 2007 video. This footage plays at full speed.

Movie S17 A downward swooping takeoff at 24:37 in the 2007 video. The view appears to be from the side. This footage plays at half speed.

Movie S18 The ventral side of the bird is visible during the vertically ascending landing that follows the downward swooping takeoff at 24:37 in the 2007 video. Just before the landing, there is a frame that reveals a dark-colored belly, light-colored underwings, a tail that projects behind the wings about the same distance as the width of the wings, and a body that has a width that is a substantial fraction of the width of the wings.

Movie S19 When the bird appears climbing after the takeoff at 24:37 in the 2007 video, there are flashes of white that are consistent of a key field mark of the Ivory-billed Woodpecker. This footage plays at half speed.

Movie S20 The first part of a downward swooping takeoff that is followed by a long horizontal glide at 2:02 in the 2007 video. The bird is hidden behind vegetation along parts of the flight path, but it is visible taking off in the upper right and then as it begins to level off in the lower left. This footage plays at half speed.

Movie S21 The second part of a downward swooping takeoff that is followed by a long horizontal glide at 2:02 in the 2007 video. The bird is hidden behind vegetation along parts of the flight path, but underwings that appear mostly white are visible in the reflection from the water. This footage plays at half speed.
